# Genome-Wide Association Study of *VKORC1* and *CYP2C9* on acenocoumarol dose, stroke recurrence and intracranial haemorrhage in Spain

**DOI:** 10.1038/s41598-020-59641-9

**Published:** 2020-02-18

**Authors:** Natalia Cullell, Caty Carrera, Elena Muiño, Nuria-Paz Torres-Aguila, Jara Cárcel-Márquez, Jonathan González-Sánchez, Cristina Gallego-Fabrega, Jessica Molina, Sarah Besora, Javier Sotoca, Maria-Teresa Buongiorno, Jordi Jiménez-Conde, Eva Giralt-Steinhauer, Reyes de Torres-Chacón, Joan Montaner, Fernando Mancha, Juan A Cabezas, Joan Martí-Fàbregas, Luis Prats-Sánchez, Pol Camps-Renom, Francisco Purroy, Serafi Cambray, María del Mar Freijo, Cristòfol Vives-Bauzá, Silvia Tur, Maria-Àngels Font, Elena López-Cancio, Maria Hernandez-Perez, Victor Obach, Ana Calleja, Juan Arenillas, Manuel Rodríguez-Yáñez, José Castillo, Tomas Sobrino, Israel Fernández-Cádenas, Jerzy Krupinski

**Affiliations:** 1 Neurology, Hospital Universitari MútuaTerrassa/Fundacio Docència i Recerca MútuaTerrassa, Terrassa, Spain; 2 Stroke Pharmacogenomics and Genetics, Biomedical Research Institute Sant Pau, Barcelona, Spain; 3 Facultat de Medicina, Universitat de Barcelona, Barcelona, Spain; 4 Neurovascular Research Laboratory, Vall d’Hebron Institute of Research (VHIR), Barcelona, Spain; 5 Centre for bioscience, School of HealthCare Science, Manchester Metropolitan University, Manchester, UK; 6 Neurology, Hospital del Mar Medical Research Institute, Barcelona, Spain; 7 Department of Neurology, Hospital Universitario Virgen Macarena, Seville, Spain; 8 Institute de Biomedicine of Seville, IBiS/Hospital Universitario Virgen del Rocío/CSIC/University of Seville & Department of Neurology, Hospital Universitario Virgen Macarena, Seville, Spain; 9 Neurology, Hospital de la Santa Creu i Sant Pau, Barcelona, Spain; 10 Stroke Unit, Department of Neurology, Universitat de Lleida, Hospital Universitari Arnau de Vilanova de Lleida, Lleida, Spain; 11 Clinical Neurosciences Group, Institut de Recerca Biomèdica de Lleida (IRBLleida), Universitat de Lleida, Lleida, Spain; 12 Neurology, Basurto Hospital, Bilbao, Spain; 13 Neurobiology Laboratory, Research Unit, Son Espases University Hospital, Illes Balears, Spain; 14 Neurology, Son Espases University Hospital, Illes Balears, Spain; 15 Neurology, Moisès Broggi Hospital, Barcelona, Spain; 16 Stroke Unit, Germans Trias i Pujol Hospital, Barcelona, Spain; 17 Stroke Unit, Hospital Universitario Central de Asturias, Asturias, Spain; 18 Neurology, Hospital Clinic, Barcelona, Spain; 19 Department of Neurology, University Clinical Hospital of Valladolid, Valladolid, Spain; 20 Clinical Neurosciences Research Laboratory, Department of Neurology, Health Research Institute of Santiago de Compostela (IDIS), Clinical University Hospital, Santiago, Spain

**Keywords:** Genome-wide association studies, Genetics of the nervous system

## Abstract

Acenocoumarol is an oral anticoagulant with significant interindividual dose variations. Variants in *CYP2C9* and *VKORC1* have been associated with acenocoumarol maintenance dose. We analysed whether any of the 49 polymorphisms in *CYP2C9* and *VKORC1* previously associated with acenocoumarol maintenance dose in a Genome-Wide Association study (GWAs) in Dutch population are associated with stroke recurrence, intracranial haemorrhage (ICH) and acenocoumarol maintenance dose in a Spanish population. We performed a GWAs using Human Core Exome-chip (Illumina) in 78 patients stroke patients treated with acenocoumarol for secondary prevention enrolled as part of the prospective investigator-initiated study (IIS) SEDMAN Study. Patients were followed-up a median of 12.8 months. Three and eight patients had recurrent stroke and ICH events, respectively. We found 14 of the 49 published variants associated with acenocoumarol maintenance dose (p < 0.05). Six polymorphisms were associated with stroke recurrence and four variants with ICH (p < 0.05). In conclusion, variants in *VKORC1* and *CYP2C9* are associated with acenocoumarol maintenance dose, stroke recurrence and ICH in a Spanish cohort. These results highlight the relevance of studying pharmacogenetics associated with efficacy and safety of anticoagulant drugs and justify studies with larger sample size and different ethnic populations.

## Introduction

Oral anticoagulants (OAs) are used for the treatment and prevention of thromboembolic diseases. Vitamin K antagonists (VKAs) are the most frequently prescribed OAs^1^, warfarin being the most used VKA worldwide. In Europe, fluindione, which is mostly prescribed in France^2^, or acenocoumarol and phenprocoumon, widely used in continental European countries^2^ are highly prescribed. They have similar properties to warfarin but a different half-life^2,3^. Direct-acting OAs (DOACs) are OAs with different targets to VKAs: rivaroxaban, edoxaban, and apixaban inhibit Xa factor, while dabigatran inhibits thrombin directly^4^. Given the different targets for OAs, personalized medicine (PM) could help in choosing the safest and most effective OA for each patient.

Despite being very effective, acenocoumarol has a narrow therapeutic window, high inter-individual variability in its pharmacokinetics and a number of drug interactions that increase the risk of new or recurrent vascular events and bleeding complications^5^. Age, sex, height, body weight, and concomitant treatments are the most relevant clinical factors associated with inter-individual acenocoumarol variation. Genetic factors are also important, mainly polymorphisms in the *VKORC1* and *CYP2C9* genes^5^, which are involved in acenocoumarol metabolism. Variants in or near these genes are associated with acenocoumarol maintenance dose, the first International Normalized Ratio (INR) result after standard dose, the time to stable dose, time in therapeutic range, and bleeding events^6^. Only one Genome-Wide association study (GWAs) investigating the association with variance of acenocoumarol maintenance dose has been performed^6^. They found association of 53 SNPs, most of them replicated in a second cohort, located in or near the *VKORC1* and *CYP2C9* genes^6^.

The frequency and effect of genetic variants is different among populations. It is important to evaluate the role of genetic variants in each specific community before clinical application. Variants on *VKORC1* and *CYP2C9* have been studied in different populations such as Russia, Chile, Serbia, and Spain, among others^5,7–9^. In Spain, some of these variants were found to be associated with time in therapeutic range, dose requirements, INR values, and risk of bleeding events in candidate gene studies^1,10,11^. However, as far as we know, no study has evaluated the role of these polymorphisms in stroke recurrence.

The objective of the present study is to evaluate whether the polymorphisms associated with acenocoumarol maintenance dose from the published GWAs^6^ are associated with stroke recurrence in a Spanish population, which has not been studied previously. Furthermore, we intend to confirm whether these variants are also associated with acenocoumarol maintenance dose and intracranial haemorrhage (ICH) occurrence. These results could address the question of whether using the right treatment for the right patient could be implemented in the Spanish population.

## Results

Sample size calculation based on the acenocoumarol GWAs^6^ showed that a minimum of 68 patients offer enough power for replication (p < 0.001) of polymorphisms associated with acenocoumarol maintenance dose. We evaluated 78 patients (44.9% men) with a median age of 79 years who were treated with acenocoumarol at a mean weekly maintenance dose of 12.25 mg/week. Patients were followed up for a median of 12.8 months. The median NIHSS at baseline and at discharge was 4.5 and 1, respectively (Table 1).

**Table 1:** Demographic description of the cohort.

N	78
**Sex**	
Men, n (%)	35 (44.9%)
Women, n (%)	43 (55.1%)
Median age, years (25–75%)	79 (70–83)
Mean dose, mg/week (SD)	12.25 (5.9)
Median follow-up, months (25–75%)	12.8 (10.64–16.79)
Median NIHSS at baseline (25–75%)	4.5 (2–7.75)
Median NIHSS at 24 hr. (25–75%)	1.5 (0–5)
Median NIHSS at discharge (25–75%)	1 (0–2)
Median CHA_2_DS_2_-VASc score (25–75%)	4 (2–5)
Median HAS-BLED score (25–75%)	2 (1–2)
HTN, n (%)	53 (67.9%)
DM, n (%)	17 (21.8%)
Smoking, n (%)	13 (16.7%)
Stroke recurrence, n	3 (3.8%)
Median time to stroke recurrence, months (25–75%)	9.79 (4.17–10.74)
ICH, n (%)	8 (10,2%)
Median time to ICH, months (25–75%)	3.94 (2.73–5.22)
Acenocoumarol discontinuation	13 (17.3%)
**Causes:**	
Labile INR, n (%)	7 (9.3%)
Stroke recurrence/bleeding event, n (%)	1 (1.3%)
Other*, n (%)	5 (6.6%)

Description of the main characteristics of the cohort included in this analysis.

NIHSS: National institute of Health Stroke Scale; HTN: Hypertension; DM: Diabetes Mellitus; ICH: Intracranial haemorrhage; INR: International normalized ratio; 25–75%: percentile 25% and percentile 75%; SD: Standard deviation.

*Other reasons for discontinuation: cancer, fractures, and unspecified reasons.

**Figure 1:**
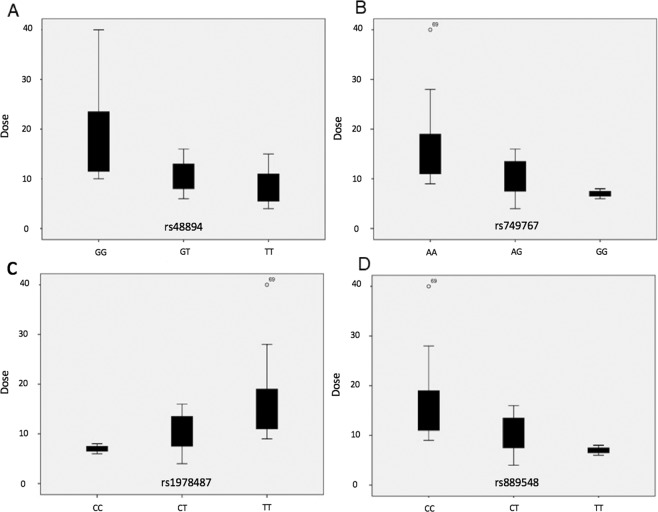
Mean dose requirements. Boxplot representing mean dose of acenocoumarol in mg/week (Y-axis) according to the genotype (X-axis) for (**A**) rs4889490, (**B**) rs749767, (**C**) rs1978487, and (**D**) rs889548.

From the 49 polymorphisms analysed, 14 SNPs were associated with acenocoumarol maintenance dose with p-values of <0.05 (Fig. 1). Four of these polymorphisms (rs1978487, rs4889490, rs749767, and rs889548) were also statistically significant when Bonferroni correction was applied. The polymorphisms rs1978487, rs749767, and rs889548 were among the top four more significant SNPs in the discovery analysis from the published GWAs^6^, with p-values of 7.82 × 10^−104^, 3.08 × 10^−103^, and 5.61 × 10^−106^, respectively (Table 2).

**Table 2:** Statistically significant polymorphisms associated with maintenance dose.

SNP	BP	CHR	effect locus	p-liter	beta-liter	beta-dose	p-dose	beta-stroke	p-stroke	beta-ICH	p-ICH	beta-comb	p-comb
rs8046001	30833321	16	*VKORC1*	7.40 × 10^−33^	2.823	**0,61**	**0,009**	**3.021**	**0,009**	1.083	0,12	**1.929**	**0,002**
rs11150596	30850242	16	*VKORC1*	1.81 × 10^−35^	2.936	**0,61**	**0,009**	**3.021**	**0,009**	1.083	0,12	**1.929**	**0,002**
rs10871454	31048079	16	*VKORC1*	2.00 × 10^−123^	−5.162	−**0,67**	**0,007**	−0,931	0,39	−**2.015**	**0,02**	−**1.668**	**0,01**
rs9933843	30903679	16	*VKORC1*	2.58 × 10^−47^	3.325	−**0,556**	**0,01**	−2.486	0,06	−1.033	0,17	−**1.820**	**0,01**
rs8058961	30809063	16	*VKORC1*	9.03 × 10^−14^	1.956	−0,323	0,24	−2.204	0,05	−1.266	0,13	−**1.639**	**0,01**
rs889548	31137712	16	*VKORC1*	5.61 × 10^−106^	−4.867	−**0,807**	**0,001**	−0,944	0,39	−**1.802**	**0,03**	−**1.601**	**0,02**
rs7197475	30642867	16	*VKORC1*	9.32 × 10^−20^	2.147	0,152	0,54	1.151	0,3	**1.703**	**0,02**	**1.473**	**0,02**
rs4889630	30877544	16	*VKORC1*	1.98 × 10^−10^	1.837	0,297	0,32	2.296	0,13	1.220	0,18	1.436	0,05
rs3747481	30666367	16	*VKORC1*	1.42 × 10^−11^	1.804	0,294	0,32	2.201	0,05	0,735	0,38	1.303	0,05
rs8058578	30726248	16	*VKORC1*	3.93 × 10^−12^	1.846	0,294	0,32	2.201	0,05	0,735	0,38	1.303	0,05
rs1978487	31129942	16	*VKORC1*	7.82 × 10^−104^	−4.827	**0,855**	**8,0 × 10**^−**04**^	0,981	0,37	1.373	0,1	1.264	0,06
rs749767	31124407	16	*VKORC1*	3.08 × 10^−103^	−4.818	−**0,813**	**0,001**	−0,954	0,39	−1.065	0,18	−1.128	0,09
rs741810	31193942	16	*VKORC1*	4.68 × 10^−19^	2.163	−0,065	0,8	−**13,63**	**0,005**	0,124	0,89	−0,912	0,21
rs8056505	31214898	16	*VKORC1*	2.49 × 10^−19^	2.178	−0,0654	0,8	−**13,63**	**0,005**	0,1239	0,89	−0,912	0,21
rs4086116	96707202	10	*CYP2C9*	3.29 × 10^−24^	−2.879	−0,311	0,26	−**11.040**	**0,04**	−0,078	0,92	−0,756	0,29
rs4917639	96725535	10	*CYP2C9*	8.02 × 10^−24^	−2.866	−0,311	0,26	−**11.040**	**0,04**	−0,078	0,92	−0,756	0,29
rs11642466	30781942	16	*VKORC1*	5.60 × 10^−14^	2.767	**0,828**	**0,01**	1.411	0,24	−0,269	0,81	0,901	0,3
rs9332169	96731310	10	*CYP2C9*	8.34 × 10^−12^	−3.442	−**0,72**	**0,03**	−11.270	0,09	−0,198	0,84	−1.021	0,31
rs10509680	96734339	10	*CYP2C9*	8.34 × 10^−12^	−3.442	−**0,72**	**0,03**	−11.270	0,09	−0,198	0,84	−1.021	0,31
rs1057910	96741053	10	CYP2C9*3	6.44 × 10^−12^	−3.459	−**0,72**	**0,03**	−11.271	0,09	−0,198	0,84	−1.021	0,31
rs9332214	96743108	10	*CYP2C9*	8.34 × 10^−12^	−3.442	**0,72**	**0,03**	11.271	0,09	−0,198	0,84	1.021	0,31
rs17790434	30520856	16	*VKORC1*	9.21 × 10^−11^	1.758	**1.528**	**0,002**	1.285	0,42	0,592	0,68	0,523	0,54
rs4889490	30823047	16	*VKORC1*	2.33 × 10^−63^	−3.802	−**0,798**	**0,001**	−1.950	0,08	−**1.644**	**0,04**	−**1.780**	**0,005**

Description of the p-values and beta-values from Teichert *et al*.^6^ (P-LITER and BETA-LITER) and from our analysis for weekly maintenance dose (P-DOSE and BETA-DOSE), risk of recurrent stroke (P-STROKE and BETA-STROKE), risk of ICH (P-ICH and BETA-ICH) and from our combined analysis for ICH events and recurrent strokes (P-COMB and BETA-COMB).

Bold numbers indicate statistically significant p-values.

We also evaluated the association of the 49 published polymorphisms with stroke recurrence and with ICH events during follow-up. Three (3.8%) and eight (10.2%) patients had a recurrent IS or ICH, respectively (Table 1). Six out of the 49 polymorphisms (rs8046001, rs4086116, rs4917639, and rs11150596) were associated with stroke recurrence. Two (rs8046001 and rs11150596) of these polymorphisms were also associated with acenocoumarol maintenance dose in our cohort (Table 2), showing association for two traits at the same time. Four variants were associated with ICH (rs4889490, rs889548, rs10871454, and rs7197475). Three of these variants (rs4889490, rs889548, and rs10871454) were associated with acenocoumarol maintenance dose in our analysis (Table 2). Moreover, the combined analysis of recurrent strokes and ICH events showed eight polymorphisms significantly associated with this outcome (Table S1). Polygenic risk scores considering the independent polymorphisms from the published GWAs for acenocoumarol maintenance dose^6^ showed an association with IS recurrence (p = 0.001) but not for ICH events nor acenocoumarol maintenance dose (p = 0.691 and 0.086, respectively).

Survival curves showed significant differences (p < 0.05) in the incidence of stroke recurrence and ICH events over time depending on rs741810 and rs10871454 genotypes, respectively. For the other polymorphisms the differences were not significant, despite some trends that could be observed in plots (Figs. S2 and S3).

## Discussion

We studied a Spanish cohort, analysing the polymorphisms previously associated with acenocoumarol maintenance dose in a previous GWAs in patients from the Netherlands^6^. The polymorphisms were located in or near the *VKORC1* and *CYP2C9* genes. Population-specific genetic properties make it necessary to perform GWAs for each population to confirm its implication for the disease.

Variations in *VKORC1* and *CYP2C9* have previously been associated with different parameters related to acenocoumarol^1^, but their implication for recurrence of IS has not been consistently elucidated and the results have been controversial. One analysis compared the use of algorithms for VKAs initial-dose guiding versus classical dosing. They did not find any effect of using genetic algorithms on a reduction in the risk of thromboembolic events^12^. However, one candidate-gene study found that variants in *VKORC1* and *CYP2C9* were associated with thrombotic events, such as stroke, TIA, and venous thromboembolism, among others^13^. Furthermore, two studies that included patients from the Chinese Han population found that *VKORC1* mutations were associated with a higher risk of cardiovascular diseases, including stroke^14,15^. Since polymorphisms in these genes are associated with less sensitivity to VKAs, patients with these variants and without a good dosage are at high risk of suffering another stroke or systemic embolism^14^. In our study, we saw that several of the variants associated with acenocoumarol maintenance dose in our cohort are also associated with the risk of suffering another stroke. Furthermore, some of the variants associated with acenocoumarol dose in the previous GWAs, are associated with recurrent stroke in our population, despite not being associated with acenocoumarol maintenance dose in our study. Moreover, the polygenic risk score based on the results from the previous GWAs highlight the importance that polymorphisms previously associated with acenocoumarol maintenance dose have also for the risk of recurrent stroke. Moreover, the lack of association (although a trend is observed) of the score with acenocoumarol maintenance dose could be related with the differences existing between populations.

Furthermore, we found polymorphisms associated with maintenance dose that are also associated with ICH events. These results show that these polymorphisms should be taken into account for acenocoumarol dosing because of their implication for the efficacy and safety of the treatment. However, the polygenic risk score obtained from previous GWAs did not show association with ICH events. One possible explanation is the lack of association previously documented^16^ for polymorphisms in *CYP2C9* and ICH. Another reason could be the different population where these variants have been studied.

Different pharmacogenetic algorithms have been developed in different populations, including the most common polymorphisms in *VKORC1* and *CYP2C9* to be used in clinical practice^1,5,17–21^. However, they do not explain all drug variability. Some of the variants found by a GWAs approach could be in these pharmacogenetic algorithms included in the future. However, it is important to find the association of genetic variants in each specific population before using genetic algorithms to ensure its plausibility in clinical practice. In this case, variants associated in a previous GWAs replicated in our Spanish cohort could be applied to the current algorithms to develop a specific algorithm designed for Spanish patients. These algorithms should focus on stroke recurrence and ICH events, two important variables from a clinical point of view. The fact that these SNPs are associated with vascular recurrence in the Spanish population could allow PM to establish the correct dose and choose an OA for which these polymorphisms are not relevant, such as dabigatran or other DOACs^22^.

Our study has some clear limitations. Mainly, the sample size is too small to find new polymorphisms associated with acenocoumarol maintenance dose in our cohort, but we calculated that it is enough to replicate some of the main candidate polymorphisms. With this sample size we have been able to detect variants in or near *VKORC1* and *CYP2C9* associated with acenocoumarol dose, stroke recurrence, and ICH in a Spanish cohort.

## Methods

Patients were included as part of the ongoing SEDMAN study (‘Dabigatran study in the early phase of stroke. New neuroimaging markers and biomarkers study’), with ClinicalTrials.gov number: NCT02742480. The SEDMAN study is a prospective, multicentre, investigator-initiated study (IIS) that consecutively enrolled stroke patients from 12 different Spanish sites from June 2016 to January 2019 (the Supplementary Material includes the detailed methodology of the study). For the present analysis, patients from the SEDMAN study who met the following inclusion criteria were analysed: cardioembolic stroke patients who initiated acenocoumarol treatment after stroke who had completed a minimum of 6 months’ follow-up. All patients or their legal representatives signed the informed consent and the project was approved by the Mútua de Terrassa Ethics Committee and then for every participating hospital. All methods were performed in accordance with the relevant guidelines and regulations for studies with human samples.

### Study endpoints

The primary endpoint of the study was the recurrence of symptomatic ischemic stroke (IS). IS diagnostic was based on neurologist criteria following physical examination and neuroimaging (computed tomography, CT or magnetic resonance imaging, MRI) in patients during treatment with acenocoumarol. We excluded patients with transient ischemic attack (TIA). We considered as IS all neurological dysfunctions produced by focal infarction observed by neuroimaging techniques and classified as cardioembolic, lacunar atherothrombotic and undetermined within the TOAST classification.

The secondary endpoints analysed were: (1) Acenocoumarol maintenance dose (considered when 3 or more INR measures ranged between 2 and 3 for 3 weeks or more^21^), and (2) Any symptomatic or asymptomatic spontaneous ICH events. Non-traumatic ICH was diagnosed when bleeding in the parenchyma (intraparenchymal haemorrhage) or the ventricular system (intraventricular haemorrhage) of patients was observed through neuroimaging techniques (CT or MRI). We excluded traumatic ICHs and (3) Combination of ICH events and recurrent strokes”.

### Follow-up

Patients were followed up using their clinical records or through telephone and clinical visits by an experienced neurologist. For the present interim analysis, a minimum of 6 months’ follow-up was considered for the recurrence and ICH registry.

### Genome-wide association study, and selection of polymorphisms

A total of 164 patients were genotyped using the Human Core Exome chip (Illumina) at Washington University (St. Louis). We performed quality controls following previous recommendations for samples and polymorphisms^23^ and we imputed the genetic variants with the Michigan Imputation Server^24^, using genotypes from 1000-Genomes Project.

From the genotyped patients, we selected 85 patients who met the inclusion criteria for this specific analysis. After quality controls, we analysed 78 patients. From all the polymorphisms imputed, we selected those associated with acenocoumarol maintenance dose in the only GWAs published in this field analysing acenocoumarol maintenance dose in Dutch population^6^. A total of 49 SNPs with significant p-values in the discovery (p < 5 × 10^−8^) and replication (p < 0.05) analyses were selected for our study (Table 2).

We have focused only on GWAs results because candidate gene studies are biased and in the case of acenocoumarol pharmacogenetics, those genes and SNPs have not been replicated or validated consistently. In contrast, GWAs are unbiased techniques with higher sample sizes and replication stages that are more effective in finding SNPs associated with a condition or disease.

### Statistical analysis

For the association analysis of polymorphisms and the different endpoints, SNPTEST v2.5.4-beta3 software was used^25^. We used the method “expected” to control genotype uncertainty. We included sex, age, and principal components 1 and 2 as covariates in the different analyses.

We calculated the sample size needed with the pwr package^26^ in R using “pwr.2p.test: two-sample proportion test” (for univariate analyses) and “pwr.f2.test: test for the general linear model” (for multivariate analyses). For the analysis of acenocoumarol maintenance dose, we considered beta values from the published GWAs^6^ and a statistical power of 0.8. For the sample size calculation needed in the pharmacogenetic analyses of stroke recurrence and ICH, we considered the number of ICH events associated with *VKORC1* genotype from Jiménez-Varo *et al*.^1^ and the total number of bleeding events associated with *CYP2C9* obtained from Visser *et al*.^27^. In the absence of articles investigating the association of these specific polymorphisms with the risk of recurrent stroke in patients treated with acenocoumarol, we assumed the mentioned calculation for ICH events as valid for stroke recurrence analysis. Using the two-sample proportion test we obtained that the minimum sample size needed to identify associations was 50 while using the test for the general linear model we obtained that the sample size needed was 81 patients.

A p-value of <0.001 was considered statistically significant after correcting for the 49 polymorphisms evaluated (Bonferroni test).

We also evaluated which SNPs were in linkage disequilibrium (R^2^ > 0.8). For the SNPs significantly associated with ICH events, rs7197475, rs4889490 and rs10871454 were independents. For the SNPs significantly associated with stroke recurrence, rs8046001, rs741810 and rs4086116 were independents and for acenocoumarol maintenance dose analysis: rs17790434, rs10871454, rs8046001, rs11642466 and rs9332169 were independents.

Moreover, we have generated weighted polygenic risk scores (GRS) based on the independent polymorphisms (R^2^ < 0.8) from the Teichert *et al*. GWAs^6^ and analysed it for association with acenocoumarol maintenance dose, ischemic stroke recurrence and ICH events. Each value was obtained as described in the Supplemental Information and in Cullell N *et al*.^28^, for weighted GRS.

### Survival analysis

We used the Survival package^29^ in R (Version 3.5.1) to perform survival analysis using Cox regression curves. We included age as a covariate in the Cox regression analysis.

## Supplementary information

Supplementary information.

## Data Availability

The data generated from this study will be made available upon request to the corresponding author.
