# Supplementary material for: Genome-Wide Association Study of VKORC1 and CYP2C9 on acenocoumarol dose, stroke recurrence and intracranial haemorrhage in Spain
Source: Sci Rep. 2020 Feb 18;10:2806. doi: 10.1038/s41598-020-59641-9 (PMC7028945; doi:10.1038/s41598-020-59641-9)
Supplement: Supplementary file 1 — Supplementary information. [file 41598_2020_59641_MOESM1_ESM.docx]

**Genome-Wide Association Study of *VKORC1* and *CYP2C9* on acenocoumarol dose, stroke recurrence and intracranial haemorrhage in Spain**

**Natalia Cullell^1,2,3^, Caty Carrera^2,4^, Elena Muiño^2^, Nuria-Paz Torres-Aguila^2^, Jara Cárcel-Márquez^2^, Jonathan González-Sánchez^1,5^, Cristina Gallego-Fabrega^1,2^, Jessica Molina^1^, Sarah Besora^1^, Javier Sotoca^1^, Maria-Teresa Buongiorno^1^, Jordi Jiménez-Conde^6^, Eva Giralt-Steinhauer^6^, Reyes de Torres-Chacón^7^, Joan Montaner^8^, Fernando Mancha^8^, Juan Caro^8^, Joan Martí-Fàbregas^9^, Luis Prats-Sánchez^9^, Pol Camps-Renom^9^, Francisco Purroy^10^, Serafi Cambray^11^, María del Mar Freijo ^12^, Cristòfol Vives-Bauzá^13^, Silvia Tur^14^, Maria-Àngels Font^15^, Elena López-Cancio^16,17^, Maria Hernandez-Perez^16^**, **Victor Obach^18^,** **Ana Calleja^19^, Juan Arenillas^19^, Manuel Rodríguez-Yáñez^20^, José Castillo^20^, Tomas Sobrino^20^, Israel Fernández-Cádenas^1,2^*, jerzy Krupinski^1,5^***

^1^Neurology. Hospital Universitari Mútua de Terrassa / Fundacio Docència i Recerca MutuaTerrassa,Spain

^2^Stroke Pharmacogenomics and Genetics, Biomedical Research Institute Sant Pau,Spain.

^3^Facultat de Medicina. Universitat de Barcelona,Spain

^4^Neurovascular Research Laboratory, Vall d'Hebron Institute of Research(VHIR), Barcelona, Spain

^5^Centre for bioscience, School of HealthCare Science, Manchester Metropolitan University, UK

^6^Neurology, Hospital del Mar Medical Research Institute,Spain.

^7^Department of Neurology, Hospital Universitario Virgen Macarena,Spain.

^8^Institute de Biomedicine of Seville, IBiS/Hospital Universitario Virgen del Rocío/CSIC/University of Seville & Department of Neurology, Hospital Universitario Virgen Macarena, Spain

^9^Neurology, Hospital de la Santa Creu i Sant Pau,Spain.

^10^Stroke Unit, Department of Neurology, Universitat de Lleida, Hospital Universitari Arnau de Vilanova de Lleida,Spain.

^11^Clinical Neurosciences Group, Institut de Recerca Biomèdica de Lleida(IRBLleida), Universitat de Lleida,Spain.

^12^Biocruces Research Institute; Cruces University Hospital. Department of Stroke.

^13^Neurobiology Laboratory, Research Unit, Hospital Universitari Son Espases,Spain

^14^Neurology, Son Espases University Hospital,Spain.

^15^Neurology, Hospital Moisès Broggi,Spain.

^16^Stroke Unit, Germans Trias i Pujol Hospital,Spain.

^17^Stroke Unit, Hospital Universitario Central de Asturias,Spain

^18^Neurology, Hospital Clinic,Spain.

^19^Department of Neurology, University Clinical Hospital of Valladolid,Spain.

^20^Clinical Neurosciences Research Laboratory, Department of Neurology, Health Research Institute of Santiago de Compostela(IDIS), Clinical University Hospital,Spain.

Corresponding Author: Jerzy Krupinski, Neurology. Hospital Universitari Mútua de Terrassa / Fundacio Docència i Recerca MutuaTerrassa, Pl.Dr.Robert 5, 08221. Terrassa,Spain

Email: jkrupinski@mutuaterrassa.cat

**Supplemental Data**

**1-Supplementary Method**

***Patients inclusion***

The Spanish sites included a part of the SEDMAN Study were: Hospital Universitari Mútua de Terrassa, Hospital del Mar, Hospital Germans Tries I Pujol, Hospital de la Santa Creu I Sant Pau, Hospital de Valladolid, Hospital Son Espases, Hospital Virgen del Rocio, Hospital de Basurto, Hospital Arnau de Vilanova, Hospital Clínic and Hospital de Santiago de Compostela. All the ethical committees from these hospitals approved the study.

The detailed inclusion criteria in the SEDMAN study was: patients ≥ 18 years old, treated with acenocoumarol or dabigatran for stroke or systemic embolism prevention following the local recommendations. All patients had a stroke or transient ischemic attack (TIA) during the previous 14 days before the initiation of anticoagulation treatment and had a diagnostic of non-valvular atrial fibrillation. Only patients with mild to moderate stroke (less than 2/3 of the vascular territory) with initial Alberta Stroke Program Early CT Score (ASPECTS) in the first CT/MRI > 6 and National Institute of Health Stroke Scale (NIHSS) < 25 were included. All patients had a general condition which allowed the 12 months’ follow-up. For this analysis, only patients treated with acenocoumarol with a minimum of 6 months-follow-up completion were included.

***GWAs analysis***

164 patients were genotyped using the Human Core Exome chip (Illumina) at Washington University in St. Louis. 499,077 single nucleotide polymorphisms (SNPs) were genotyped.

We performed quality controls following previous recommendations for samples and polymorphisms^1^ using PLINK^2^ (http://zzz.bwh.harvard.edu/plink/), QCTOOL (https://www.well.ox.ac.uk/~gav/qctool_v1/#overview) and GTOOL V.0.7.5 (http://www.well.ox.ac.uk/%7ecfreeman/software/gwas/gtool.html) software.

Sample quality controls consist on: heterozygosity (>-0.3), missingness (<0.05), identity by state (>0.18) and removal of discordant sex patients. Principal component analysis was used to identify population subgroups. SNPs quality controls were: missingness (<0.05), minor allele frequency (MAF) (>0.01), failure of Hardy-Weinberg equilibrium SNPs.

We imputed the genetic variants with the Michigan Imputation Server ^3^, using genotypes from 1000 Genomes Project. After imputation and quality controls (removal of polymorphisms with maf < 0.01 and imputation information < 0.5) we obtained information from 13,933,055 SNPs for our analyses.

***GRS construction***

We constructed a polygenic risk score based on the beta values from Teichert et al. ^4^ from the independent polymorphisms (R^2^ < 0.8) from their paper. Then, we obtained the risk allele dosage (0,1 or 2) per patient for each polymorphism and multiplied this value for the beta value. Then, we summed this values to obtain a single result per patient. Finally, we applied t-test for the association analysis of the score with ICH events and stroke recurrence and Pearson correlation for the association of the score with acenocoumarol maintenance dose.

**2- Supplemental Tables**

**Table S1:** Associations for the significant 49 polymorphisms in Teichert et al. GWAs ^4^ in the combined analysis of ICH events and recurrent stroke.

| **SNP** | **BP** | **CHR** | **effect locus** | **p-liter** | **beta-liter** | **beta-combined** | **p-combined** |
| --- | --- | --- | --- | --- | --- | --- | --- |
| **rs1978487** | 31129942 | 16 | *VKORC1* | 7.82x10^-104^ | -4.827 | 1.264 | 0.06 |
| **rs4889490** | 30823047 | 16 | *VKORC1* | 2.33x10^-63^ | -3.802 | **-1.780** | **0.005** |
| **rs749767** | 31124407 | 16 | *VKORC1* | 3.08x10^-103^ | -4.818 | -1.128 | 0.09 |
| **rs889548** | 31137712 | 16 | *VKORC1* | 5.61x10^-106^ | -4.867 | **-1.601** | **0.02** |
| **rs17790434** | 30520856 | 16 | *VKORC1* | 9.21x10^-11^ | 1.758 | 0.523 | 0.54 |
| **rs10871454** | 31048079 | 16 | *VKORC1* | 2.00x10^-123^ | -5.162 | **-1.668** | **0.01** |
| **rs8046001** | 30833321 | 16 | *VKORC1* | 7.40x10^-33^ | 2.823 | **1.929** | **0.002** |
| **rs11150596** | 30850242 | 16 | *VKORC1* | 1.81x10^-35^ | 2.936 | **1.929** | **0.002** |
| **rs11642466** | 30781942 | 16 | *VKORC1* | 5.60x10^-14^ | 2.767 | 0.901 | 0.30 |
| **rs9933843** | 30903679 | 16 | *VKORC1* | 2.58x10^-47^ | 3.325 | **-1.820** | **0.01** |
| **rs9332169** | 96731310 | 10 | *CYP2C9* | 8.34x10^-12^ | -3.442 | -1.021 | 0.31 |
| **rs10509680** | 96734339 | 10 | *CYP2C9* | 8.34x10^-12^ | -3.442 | -1.021 | 0.31 |
| **rs1057910** | 96741053 | 10 | *CYP2C9*3* | 6.44x10^-12^ | -3.459 | -1.021 | 0.31 |
| **rs9332214** | 96743108 | 10 | *CYP2C9* | 8.34x10^-12^ | -3.442 | 1.021 | 0.31 |
| **rs8058961** | 30809063 | 16 | *VKORC1* | 9.03x10^-14^ | 1.956 | **-1.639** | **0.01** |
| **rs4086116** | 96707202 | 10 | *CYP2C9* | 3.29x10^-24^ | -2.879 | -0.756 | 0.29 |
| **rs4917639** | 96725535 | 10 | *CYP2C9* | 8.02x10^-24^ | -2.866 | -0.756 | 0.29 |
| **rs4889630** | 30877544 | 16 | *VKORC1* | 1.98x10^-10^ | 1.837 | 1.436 | 0.05 |
| **rs3747481** | 30666367 | 16 | *VKORC1* | 1.42x10^-11^ | 1.804 | 1.303 | 0.05 |
| **rs8058578** | 30726248 | 16 | *VKORC1* | 3.93x10^-12^ | 1.846 | 1.303 | 0.05 |
| **rs7197475** | 30642867 | 16 | *VKORC1* | 9.32x10^-20^ | 2.147 | **1.473** | **0.02** |
| **rs741810** | 31193942 | 16 | *VKORC1* | 4.68x10^-19^ | 2.163 | -0.912 | 0.21 |
| **rs8056505** | 31214898 | 16 | *VKORC1* | 2.49x10^-19^ | 2.178 | -0.912 | 0.21 |

Description of the p-values and beta-values from Teichert et al.^4^ (P-LITER and BETA-LITER) and from our combined analysis for ICH events and recurrent strokes (P-COMB and BETA-COMB).

Bold numbers indicate statistically significant p-values.

**3- Supplemental figures**

**Figure S1:** Flowchart for the GWAs quality controls (QCs)


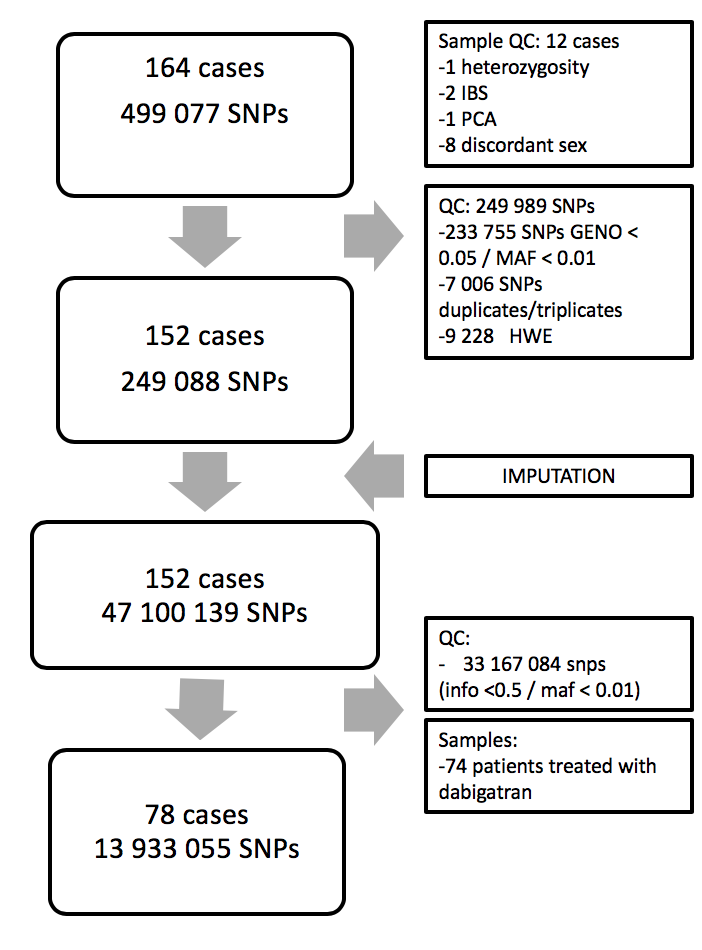


IBS: identity by state; PCA: principal components; HWE: Hardy Weinberg equilibrium; maf: minor allele frequency

**
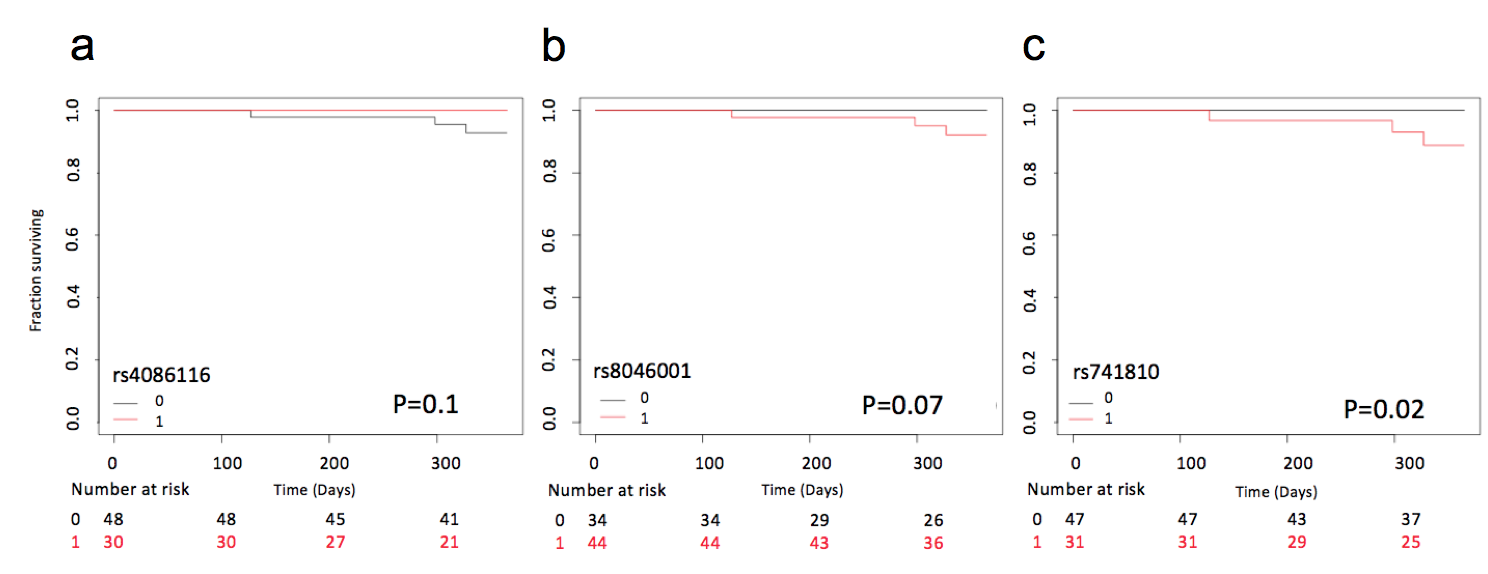
Figure S2:** Survival curves for recurrent stroke

Plots for significant and independent SNPs associated with recurrent stroke. The Y-axis shows the proportion surviving and the X-axis shows the number of days stratified by a) rs4086116, b) rs741810, and c) rs8046001

**
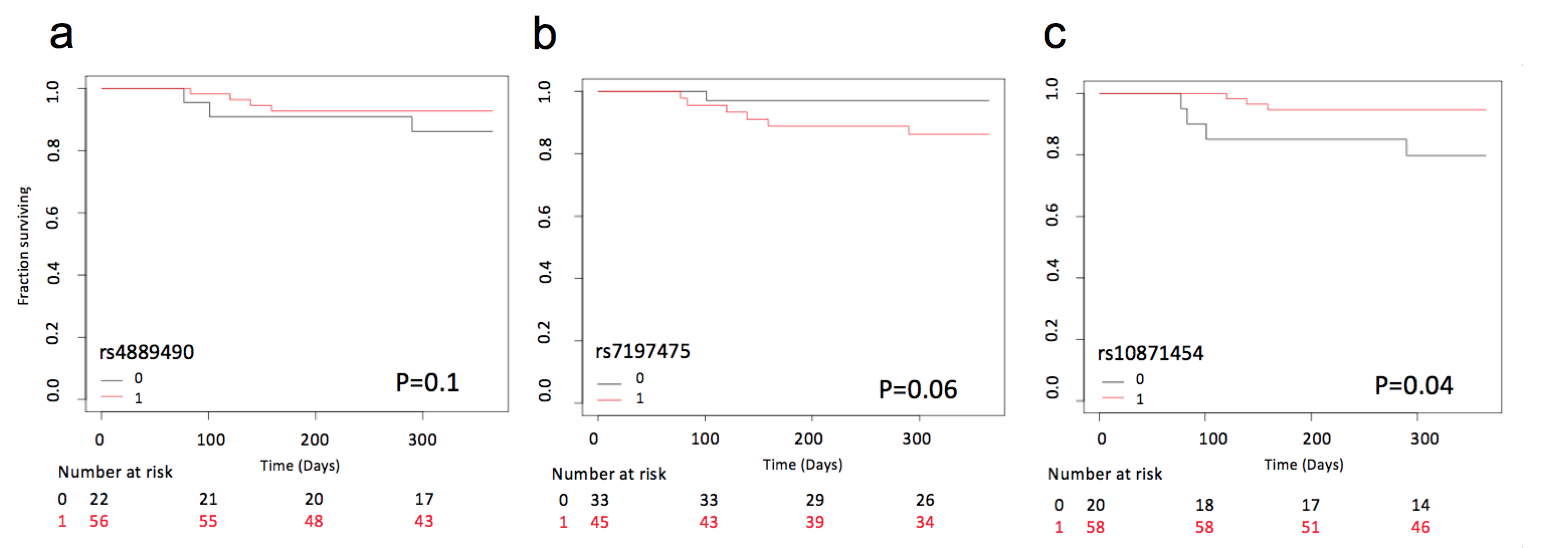
Figure S3:** Survival curves for ICH

Plot for significant and independent SNPs (adjusted by age) associated with ICH. The Y-axis shows the proportion surviving and the X-axis shows the number of days stratified by a) rs7197474, b) rs4889490, and c) rs10871454

**REFERENCES**

1. Malik R, Chauhan G, Traylor M, et al. Multiancestry genome-wide association study of 520,000 subjects identifies 32 loci associated with stroke and stroke subtypes. *Nat Genet* 2018; 50: 524–537.

2. Purcell S, Neale B, Todd-Brown K, et al. PLINK: a tool set for whole-genome association and population-based linkage analyses. *Am J Hum Genet*. Epub ahead of print 2007. DOI: 10.1086/519795.

3. Das S, Forer L, Schönherr S, et al. Next-generation genotype imputation service and methods. *Nat Genet* 2016; 48: 1284–1287.

4. Teichert M, Eijgelsheim M, Rivadeneira F, et al. A genome-wide association study of acenocoumarol maintenance dosage. *Hum Mol Genet* 2009; 18: 3758–68.
